# The effect of government publicity and guidance on farmers’ ecological environment governance participation behavior—The mediating effect of environmental literacy and perceived value

**DOI:** 10.1371/journal.pone.0328274

**Published:** 2025-07-23

**Authors:** Haoran Wei, Juan Yao, Fuhong Wang

**Affiliations:** 1 College of Economics and Management, Xinjiang Agricultural University, Urumqi, Xinjiang Uygur Autonomous Region, China; 2 College of Agriculture, Forestry Economics and Management, Lanzhou University of Finance and Economics, Lanzhou, Gansu Province, China; Huaqiao University, CHINA

## Abstract

As an important policy tool for rural ecological environment governance, it is crucial to clarify the mechanism through which government publicity and guidance affects farmers’ participation behavior, thereby enhancing the effectiveness of rural ecological governance. Based on survey data from rural households in Changji Prefecture, Xinjiang, this study empirically examines the influence of government publicity and guidance on farmers’ decision-making behavior, protective behavior, and supervisory behavior related to ecological environment governance, employing an Ordered Probit model, OLS regression, and a Generalized Structural Equation Model. The findings indicate that: (1) Government publicity and guidance, Environmental literacy, and Perceived Value exert significant positive effects on farmers’ multidimensional ecological environment governance behaviors; (2) Heterogeneity analysis reveals that these three factors have a stronger promoting effect on the ecological environment governance behaviors of low-income farmers; (3) Environmental literacy and Perceived Value serve as significant mediators in the relationship between government publicity and guidance and farmers’ ecological environment governance participation behavior, and two together construct a chain mediation path of “government publicity and guidance-environmental literacy-perceived value-ecological and environmental governance behavior”. Based on these findings, this study proposes policy recommendations including refining government publicity and guidance strategies, systematically enhancing environmental literacy, facilitating the internalization of Perceived Value, and implementing differentiated policy support mechanisms.

## 1 Introduction

In many developing countries, agricultural modernization and rural economic growth have long relied on a resource-intensive and unsustainable development model [1,2]. While this model has temporarily boosted agricultural output and farmers’ income, it has also resulted in a series of negative externalities—including ecosystem degradation, excessive resource consumption, and environmental pollution—thereby exacerbating rural ecological deterioration, undermining farmers’ health and well-being, and imposing systemic constraints on the sustainable development of rural socio-economic systems [3]. To address these challenges, many governments have gradually developed integrated rural ecological governance frameworks that encompass legal regulations, fiscal incentives, technological support, and other complementary measures. However, the actual effectiveness of these governance systems remains unsatisfactory, primarily due to the excessive centralization of responsibilities within governmental agencies and the insufficient engagement of farmers, resulting in a persistent governance dilemma characterized by “government-led efforts and passive farmer participation” [4]. As key actors in rural ecological environment governance, farmers are both potential contributors to environmental degradation and direct beneficiaries of governance outcomes. Their behavioral choices influence both the stability of local ecosystems and the implementation of governance policies, thereby shaping the long-term sustainability of rural ecological transformation [5]. Therefore, identifying effective strategies to stimulate farmers’ governance behavior, shift their roles from passive recipients to active participants, and foster a multi-actor, collaborative governance model has become a central challenge in global rural ecological governance practices.

In recent years, the mechanisms underlying farmers’ behavioral responses within ecological environment governance have emerged as a focal point in environmental social science and sustainable agriculture research [6,7]. Farmers’ ecological environment governance participation behavior encompasses not only their willingness and actual engagement in various environmental governance projects but also their multilayered interactions in environmental information acquisition, policy comprehension, process-level supervisory behavior, and feedback delivery [8]. Scholars generally categorize these behaviors into three core dimensions: Decision-making behavior, referring to whether farmers express opinions or participate in rule-making through platforms such as village governance design and public consultation; Protective behavior, referring to farmers’ proactive participation in eco-practices like waste sorting, wastewater treatment, and pollution control; and Supervisory behavior, which reflects farmers’ roles in monitoring and reporting violations or evaluating policy implementation [9]. Regarding the behavioral drivers, prior research has identified both internal and external determinants. Internal factors such as age, education, income level, and environmental cognition have been shown to influence farmers’ decision-making behavior to varying extents [9–12]. Externally, financial subsidies, collective action mechanisms, local mobilization, and legal frameworks serve as important incentives and constraints that shape environmental actions [13–19]. Moreover, embedded social structures such as social capital and neighborhood relations facilitate information dissemination and behavioral imitation, providing a key social foundation for farmers’ environmental behaviors [20]. Despite offering a robust body of theoretical and empirical evidence, current literature still faces notable limitations. First, most studies focus primarily on general participation, while overlooking the more nuanced dimensions of decision-making and supervisory behavior. Second, although government publicity and guidance has been widely utilized as a non-compulsory and non-material policy tool in rural governance, its behavioral impact and mechanisms remain underexplored both theoretically and empirically. Third, existing research tends to emphasize institutional incentives as dominant explanatory factors, often neglecting the interaction between internal cognitive structures and external policy stimuli. The absence of an integrated framework that links internal cognition with institutional context makes it difficult to fully capture how policy messages are perceived, internalized, and translated into concrete behavioral responses..

Changji City, located in Changji Hui Autonomous Prefecture, Xinjiang, is situated in the central segment of the economic belt along the northern slope of the Tianshan Mountains, covering approximately 8,215 square kilometers, and represents a prototypical oasis agricultural region in western China. The area is characterized by coexisting challenges such as water scarcity, intensive agricultural activity, and fragile ecosystems, exhibiting structural parallels with many developing regions worldwide in terms of resource endowment, environmental pressures, and governance constraints. In recent years, Changji has served as an exemplary case for investigating the interaction between government publicity and guidance and farmers’ behavioral responses, particularly as a pilot region for national ecological civilization, green transition, and rural revitalization initiatives. Against this backdrop, the present study takes rural Changji as its empirical focus, draws upon household-level microdata, and employs an Ordered Probit model to systematically investigate how government publicity and guidance shapes farmers’ ecological environment governance participation behavior. It further explores the potential mediating roles of Environmental literacy and Perceived Value. The main marginal contributions of this study are threefold: First, it develops a multidimensional analytical framework encompassing Decision-making behavior, Protective behavior, and Supervisory behavior, thus addressing the gap in existing literature that tends to focus on single behavioral dimensions. Second, by taking government publicity and guidance as a representative non-coercive policy tool, the study systematically examines its motivational effects in grassroots ecological governance, thereby enriching theoretical perspectives on the behavioral impact of policy instruments. Third, the research integrates external policy intervention with internal cognitive mechanisms by introducing Environmental literacy and Perceived Value as mediators, and proposes a three-stage influence mechanism—“Government publicity and guidance – Cognitive structure – Behavioral response”—to deepen our understanding of farmers’ ecological behavior formation processes in developing regions.

## 2 Theoretical analysis and research hypotheses

**Fig 1:**
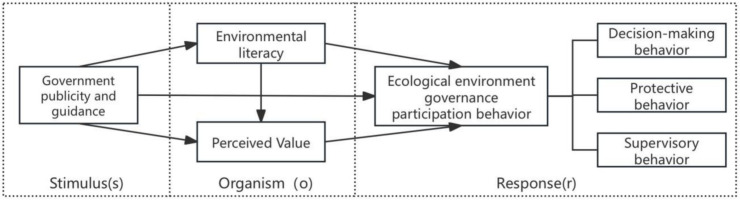
Mechanism diagram of the influence of government guidance, environmental literacy and value perception on farmers’ ecological environment governance behavior based on SOR theory.

The Stimulus–Organism–Response (SOR) theory, initially proposed by Mehrabian and Russell, is designed to explain how individuals experience psychological changes and subsequently exhibit behavioral responses when exposed to external environmental stimuli [21]. The theory posits that external stimuli (S) activate internal organismic responses (O)—encompassing cognitive, emotional, and physiological dimensions—which in turn elicit corresponding behavioral responses (R) [22]. Within this theoretical framework, farmers’ ecological environment governance participation behavior is shaped both by external stimuli originating from governmental and societal sources, and by internal psychological attributes such as cognition and motivation. The influence of external stimuli and intrinsic factors on farmers’ behavior is not mutually exclusive; rather, external stimuli may exert indirect effects by modifying farmers’ internal constructs, including cognition, attitudes, and values [23].This conceptual pathway is illustrated in Fig 1, which presents the mechanism through which government publicity and guidance influence farmers’ ecological environment governance behavior via the mediating roles of environmental literacy and perceived value.

### 2.1 Influence of government publicity and guidance, environmental literacy and perceived value on farmers’ ecological environment governance participation behavior

Government publicity and guidance refers to the process through which governmental or public institutions disseminate information related to policies, public affairs, and societal concerns to the public via multiple communication channels [24]. As an informal external stimulus, government publicity and guidance can exert significant influence on farmers’ ecological environment governance participation behavior by means of information dissemination, incentive provision, and regulatory enforcement [25]. Specifically, with respect to decision-making behavior, government publicity and guidance enhances farmers’ comprehension and acceptance of ecological policies by highlighting benefits such as improved arable land quality, increased agricultural output, and financial support, thereby stimulating their engagement in expressing opinions and participating in rule-making processes within village governance design and consultation platforms. In the realm of protective behavior, government publicity and guidance can promote model cases of green agriculture, disseminate environmental knowledge, and provide technical training to raise farmers’ awareness of ecological protection. This, in turn, encourages them to adopt eco-friendly practices such as film recycling, soil testing and formula fertilization, garbage sorting, and wastewater treatment. Regarding supervisory behavior, government publicity and guidance strengthens farmers’ understanding of the importance of ecological management, enhances their problem awareness and civic responsibility, and encourages their involvement in overseeing the implementation of ecological governance projects. By establishing participatory monitoring mechanisms—such as involving farmers in project evaluation and operational oversight—the government can further enhance their supervisory motivation and improve governance transparency and effectiveness. Based on this, the following hypotheses are proposed:

H1: Government publicity and guidance has a significant positive effect on farmers’ ecological environment governance participation behavior.

Environmental literacy refers to the integrated expression of individuals’ environmental knowledge, value orientations, and behavioral competencies in the field of environmental protection [26]. In the context of rural ecological environment governance, enhanced environmental literacy equips farmers with greater knowledge reserves and stronger behavioral motivations, thereby increasing their initiative and enthusiasm for governance participation [27]. Specifically, with respect to decision-making behavior, farmers with higher levels of environmental literacy are better able to comprehend the goals and implications of ecological policies, strengthen their identification with relevant policies, and actively engage in the design of village-level governance systems and formulation of ecological rules. In terms of protective behavior, environmental education and knowledge accumulation enable farmers to deepen their understanding of sustainable agriculture and land conservation, enhance their sense of environmental responsibility, and promote the adoption of environmentally friendly practices such as green planting and water-saving irrigation—thereby contributing to agricultural green transformation and ecological improvement. Regarding supervisory behavior, farmers with higher environmental literacy are more proactive in identifying issues and deficiencies in ecological governance, offer constructive feedback, and participate in governance assessments to ensure the effective implementation and continuous refinement of governance measures. In addition, their deeper environmental cognition and value alignment further motivate them to assume more active social roles in project supervision, thereby forming a critical grassroots force for ecological governance. Based on this, the following hypotheses are proposed:

H2: Environmental literacy has a significant positive effect on farmers’ ecological environment governance participation behavior.

Perceived Value refers to an individual’s subjective evaluation of the benefits, returns, and overall significance of a given behavior or activity [28]. In the context of rural ecological environment governance, the perceived value of such participation behavior directly influences farmers’ motivations and behavioral responses, particularly as they are viewed as “rational economic agents” [29]. Specifically, regarding decision-making behavior, a higher level of perceived value reinforces farmers’ understanding of the strategic importance of ecological governance and facilitates their recognition of long-term economic gains and collective benefits derived from governance participation, thus encouraging their active involvement in policy development and grassroots implementation. With respect to protective behavior, farmers with higher Perceived Value are more aware of the direct economic returns associated with ecological practices, such as improved soil fertility, increased yields, and access to government subsidies, and are more inclined to align ecological practices with their own interests, thereby increasing their willingness and frequency to adopt green agricultural technologies and engage in environmental protection practices. Regarding supervisory behavior, Perceived Value strengthens farmers’ willingness to engage in oversight by increasing their expectations and concerns regarding governance effectiveness. When farmers perceive that ecological governance outcomes contribute to improved quality of life and public welfare within the community, they are more likely to proactively participate in project monitoring, feedback, and evaluation, thereby promoting transparency and the continuous optimization of governance implementation. Based on this, the following hypotheses are proposed:

H3 Perceived Value has a significant positive effect on farmers’ Ecological environment governance participation behavior.

### 2.2 The mediating role of Environmental literacy and Perceived Value in Government publicity and guidance in influencing farmers’ Ecological environment governance participation behavior

According to the SOR theory, external stimuli can indirectly influence individual behavioral responses by acting upon internal psychological mechanisms [21,22]. Under this theoretical framework, government publicity and guidance not only exert a direct influence on farmers’ ecological governance behavior but also operate indirectly by enhancing their environmental literacy [30]. Specifically, the government disseminates environmental knowledge through multiple communication channels, thereby strengthening farmers’ ecological awareness and stimulating their participation in ecological environment governance behavior. Environmental literacy encompasses not only knowledge of ecological issues but also pro-environmental attitudes and the capacity to implement sustainable practices. Government publicity and guidance serves to reinforce farmers’ sense of social responsibility and environmental commitment by highlighting the urgency and public value of ecological protection, thus encouraging their active engagement in ecological governance efforts. Based on the above analysis, this study proposes the following hypotheses:

H4: Environmental literacy plays a positive mediating role between government publicity and guidance and farmers’ ecological environment governance participation behavior.

Perceived Value, as a key internal driver of farmers’ Ecological environment governance participation behavior, is likely to serve as a significant positive mediator in the relationship between government publicity and guidance and farmers’ engagement in ecological governance. Specifically, by communicating the multifaceted value of ecological governance and its long-term benefits, government publicity and guidance enables farmers to recognize that ecological protection entails not only environmental responsibility but also tangible economic returns, such as land appreciation and agricultural market premiums [31]. Accordingly, the greater farmers’ Perceived Value of ecological governance, the stronger their initiative and motivation to engage in governance practices. Thus, government publicity and guidance promotes farmers’ governance participation by enhancing their perceived value of ecological governance, thereby establishing a mediating pathway between government publicity and guidance, perceived value, and ecological environment governance participation behavior. Based on this, this study proposes the following hypotheses:

H5: Perceived Value plays a positive mediating role between government publicity and guidance and farmers’ ecological environment governance participation behavior.

Environmental literacy not only directly influences farmers’ ecological environment governance participation behavior, but also indirectly shapes their decision-making by enhancing Perceived Value. Specifically, farmers with higher environmental literacy typically possess stronger environmental knowledge and ecological responsibility awareness, enabling them to better recognize the multidimensional economic, social, and environmental value of ecological governance behaviors [30]. This enhanced cognitive capacity substantially increases their Perceived Value of the long-term benefits and public welfare associated with ecological governance. Moreover, environmental literacy manifests not only as knowledge acquisition, but also as the internalization of emotional commitment and behavioral responsibility [26]. Farmers with high environmental literacy tend to possess a stronger environmental mission, perceiving ecological governance participation as a personal social responsibility, which further strengthens their recognition of the environmental and social values embedded in policy measures [27]. Based on the above analysis, government publicity and guidance can effectively enhance farmers’ environmental literacy, while perceived value serves as a key psychological bridge between internal cognition and behavioral responses. It can therefore be inferred that government publicity and guidance enhances farmers’ environmental literacy, which in turn increases their perceived value of ecological governance, ultimately promoting their active participation in ecological environment governance participation behavior. Based on this, the following hypotheses are proposed in this study:

H6: Environmental literacy and Perceived Value play a positive chain mediating role between government publicity and guidance and farmers’ ecological environment governance participation behavior.

## 3 Data sources and research methodology

### 3.1 Data sources

The data used in this study were obtained through field research conducted across six townships—Erliugong, Yushugou, Daxiqu, Liugong, Binhu, and Dudumba—in Changji City, Xinjiang, encompassing 30 administrative villages. The research was conducted in two stages. The first phase, in March 2021, involved interviews with township government personnel to obtain baseline information for questionnaire design. The second phase, conducted in January 2022, consisted of household-level surveys targeting the household head or primary agricultural laborer in households with farming experience. To ensure data authenticity and representativeness, the survey was conducted via face-to-face interviews. A total of 320 questionnaires were distributed, and 315 valid responses were obtained after removing incomplete or logically inconsistent entries, yielding a 98.44% valid response rate. The sample structure reveals that 63.2% of respondents were male, with most aged 41 years and above. While most households reported satisfactory health conditions, their annual income levels were generally low, with 90.1% of households reporting an income below 100,000 yuan. Overall, the sample captures the typical characteristics of farming households in agricultural regions of western China, demonstrating strong representativeness and analytical value (**Table 1**).

**Table 1:** Basic information of the sample farmers.

Variable	Category	Frequency (n)	Percentage (%)	Variable	Category	Frequency (n)	Percentage (%)
Gender	Male	199	63.2%	Political party member	Non-member	227	72.1%
	Female	116	36.8%		Member	88	27.9%
Age group	18-30 years	2	0.6%	Cropland quality	Very poor	6	1.9%
	31-40 years	26	8.3%		Poor	35	11.1%
	41-50 years	94	29.8%		General	136	43.2%
	51-60 years	121	38.4%		Good	113	35.9%
	60 years and above	72	22.9%		Excellent	25	7.9%
Health status	Very poor	3	1.0%	Gross household income	Less than ¥50,000	156	49.5%
	Poor	23	7.3%		¥50,000–100,000	128	40.6%
	General	59	18.7%		¥100,000–150,000	11	3.5%
	Good	164	52.1%		¥160,000–200,000	8	2.5%
	Excellent	66	21.0%		More than ¥200,000	12	3.8%

### 3.2 Variable Selection and Descriptive Selection

(1) The explanatory variable in this study is the farmers’ ecological environment governance participation behavior. To comprehensively capture its multidimensional nature, the behavior was subdivided into three categories: Decision-making behavior, Protective behavior, and Supervisory behavior. Each behavioral dimension was measured by four corresponding survey items. Participation was scored on a scale from 0 to 4, with 0 indicating no participation and 4 indicating engagement in all four activities. This scoring system was used to quantify the degree of farmers’ involvement in ecological governance practices.
(2) The explanatory variable is government publicity and guidance. This variable was designed to capture the frequency with which farmers perceive government publicity and guidance efforts, as well as the extent of its informational coverage, in order to evaluate its effectiveness in incentivizing behavioral responses. The specific measurement item was “Frequent publicity and guidance on ecological management,” and farmers’ perceived attitudes were quantitatively assessed using a five-point Likert scale.
(3) The mediating variables in this study are Environmental literacy and Perceived Value. According to relevant theories in environmental behavior studies, environmental literacy is not merely an accumulation of knowledge about environmental issues, but also encompasses a sense of responsibility toward ecological protection and the practical ability to address environmental challenges [32]. Therefore, this study categorizes environmental literacy into three dimensions: environmental cognition, environmental responsibility, and environmental knowledge and skills [33]. Specifically, environmental cognition assesses the level of farmers’ understanding and awareness of ecological issues and their governance outcomes; environmental responsibility reflects farmers’ moral obligation in pro-environmental behavior, emphasizing their internalized sense of duty toward ecological protection; and environmental knowledge and skills evaluate farmers’ capacity to solve environmental problems and their practical proficiency, such as the adoption of green agricultural technologies and environmentally friendly production practices [34]. The construction of Perceived Value is based on the Perceived Value Theory, which posits that an individual’s motivation to engage in a particular behavior stems from their perception of the potential returns associated with that behavior. These returns typically include economic benefits, social recognition, and ecological improvement [35]. Accordingly, this study divides Perceived Value into three dimensions: economic value, ecological-environmental value, and social value. Economic value reflects the material benefits that farmers may obtain from participating in ecological environment governance, such as land value appreciation, government subsidies, and increased agricultural output; ecological-environmental value captures farmers’ recognition of improvements in the ecological environment and their expectations for sustainable development; and social value refers to the social recognition, sense of responsibility, and enhancement of collective well-being that farmers gain through their involvement in ecological governance [36]. Both mediating variables were measured using a five-point Likert scale, and the entropy weighting method was employed to determine the weights of each dimension. Composite scores were then calculated accordingly, as shown in Table 2.

**Table 2:** The results of the assignment of the indicators of the dimensions of Environmental literacy and Perceived Value.

Construct	Dimension	Norm	Mean	Standard Deviation	Entropy Weight
Environmental Literacy	Environmental Cognition	Ecological governance can mitigate natural resource degradation: Strongly disagree = 1; Disagree = 2; Fairly = 3; Agree = 4; Strongly agree = 5	4.4889	0.5938	0.1499
	Environmental Responsibility	You can feel guilty about over-fertilizing: strongly disagree = 1; disagree = 2; generally = 3; agree = 4; strongly agree = 5	4.2317	0.7739	0.1547
	Environmental Knowledge & Skills	Problems with farmland ecosystems can be detected in a timely manner: Strongly Disagree = 1; Disagree = 2; Fair = 3; Agree = 4; Strongly Agree = 5	3.3429	1.0482	0.6954
Perceived Value	Economic Value	The quality of your family’s produce has improved compared to three years ago: strongly disagree = 1; disagree = 2; fair = 3; agree = 4; strongly agree = 5	3.8984	0.7712	0.0182
	Environmental Value	Do you think the ecological environment of the village has been improved: strongly disagree = 1; disagree = 2; generally = 3; agree = 4; strongly agree = 5	4.0317	0.8553	0.2493
	Social Value	Participation in ecological governance has deepened the democratic management of the village: strongly disagree = 1; disagree = 2; generally = 3; agree = 4; strongly agree = 5	3.2190	1.0373	0.7325

(4) Control Variables. To control for potential confounding effects in the estimation, a broad set of control variables was included, covering household demographics, social identity, and agricultural production conditions, specifically including: gender and age (basic demographic attributes); health status (physical capacity for participation); literacy level (ability to understand policies and environmental cognition); party membership and cadre status (institutional participation and social influence); membership in professional farmers’ cooperatives (degree of social resource embeddedness); and quality of arable land and number of agricultural laborers (agricultural production capacity). Controlling for these variables allows for a more accurate estimation of the true effects of government publicity and guidance and the mediating variables on farmers’ ecological environment governance participation behavior. Variable coding and descriptive statistics are presented in Table 3.

**Table 3:** Description of relevant variables and descriptive statistics.

Variant	Variable type		Meaning and Assignment	Mean	Std.
explanatory variable	Government publicity and guidance		Awareness of government efforts in ecological publicity. 1 = Strongly disagree to 5 = Strongly agree.	3.9302	0.9618
Explanatory	Farmers’ Ecological environment governance participation behavior	Decision-making behavior	Number of participants in the development of ecological governance systems, program development, construction of facilities, and use of funds	0.9682	1.3089
		Protective behavior	Number of participants in water-saving irrigation, soil-formulated fertilizer application, effective use of straw, and recycling of agricultural films	1.9905	1.9976
		Supervisory behavior	Number of Participants in Ecological Governance Decision-making Supervision, Funding Supervision, Behavioral Supervision, and Pollution Supervision	1.0031	1.3581
Intermediary	Environmental literacy		Weighted composite score of environmental awareness, responsibility, and skills.	0.7016	0.2270
	Perceived Value		Weighted composite score of perceived economic, environmental, and social value.	0.5884	0.2289
Control	Gender		Female = 0, Male = 1	0.3683	0.4831
	Age		18-30 = 1; 31-40 = 2; 41-50 = 3; 51-60 = 4; 60+ = 5	3.7460	0.9233
	Health status		Very poor = 1; Poor = 2; Fair = 3; Good = 4; Excellent = 5	3.8476	0.8682
	Education level		Below elementary school = 1; middle school = 2; high school/secondary school = 3; bachelor’s degree/college = 4; master’s degree and above = 5	1.5873	0.6343
	Party member		Non-member = 0; member = 1	0.2794	0.4494
	Village cadre		Non-village cadre = 0; village cadre = 1	0.0444	0.2064
	Cooperative membership		0 = Not a member; 1 = Member	0.0857	0.2804
	Cropland quality		Very poor = 1; Poor = 2; Fair = 3; Good = 4; Excellent = 5	3.3683	0.8545
	Agricultural labor force		Number of family agricultural laborers	2.0032	0.5442

### 3.3 Model construction

#### 3.3.1 Ordered Probit Models.

The core independent variable in this study is the level of government publicity and guidance, while the dependent variable is farmers’ ecological environment governance participation behavior, which is further disaggregated into decision-making behavior, protective behavior, and supervisory behavior. Each behavior variable is assigned a value from 0 to 4, representing increasing levels of participation intensity, making it an ordered categorical variable. Accordingly, the Ordered Probit model is employed for estimation and analysis. The basic regression model is as follows:


$$Behavior_{i}^{*}=\beta Guide_{i}+\gamma Controls_{i}+\varepsilon_{i}$$ (1)

$$Behavior_{i}=\{\begin{array}{c} {}*20l0,Behavior_{i}^{*}\leq \gamma_{0}\\ 1,\gamma_{0}<Behavior_{i}^{*}\leq \gamma_{1}\\ 2,\gamma_{1}<Behavior_{i}^{*}\leq \gamma_{2}\\ 3,\gamma_{2}<Behavior_{i}^{*}\leq \gamma_{3}\\ 4,Behavior_{i}^{*}>\gamma_{3} \end{array}$$ (2)

Where$Behavior_{i}^{*}$ denotes the potential tendency of farmers’ Ecological environment governance participation behavior;$Guide_{i}$ denotes the intensity of government publicity and guidance;$Controls_{i}$ denotes the control variable;$\varepsilon_{i}$ denotes the random error term;$\gamma_{0}$,$\gamma_{1}$,$\gamma_{2}$,$\gamma_{3}$ denote the estimated split points.

#### 3.3.2 Models of mediating effects.

In order to further reveal the intrinsic mechanism of government publicity and guidance affecting farmers’ ecological environment governance participation behavior, this study introduces two mediating variables, environmental literacy and perceived value, and constructs a multiple mediation and chain mediation model. Given that the dependent variables and mediator variables are all ordered categorical variables, this study uses generalized structural equation modeling to estimate the mediation effects.

(1) single-mediation effect model (SME)

$$Behavior_{i}=c_{1}\cdot Guide_{i}+\alpha_{1}\cdot Controls_{i}+\mu_{1}$$ (3)

$$M_{i}=a\cdot Guide_{i}+\alpha_{2}\cdot Controls_{i}+\mu_{2}$$ (4)

$$Behavior_{i}=c_{2}\cdot Guide_{i}+b\cdot M_{i}+\alpha_{3}\cdot Controls_{i}+\mu_{3}$$ (5)

where *M*_*i*_ is the mediating variable (Environmental literacy or Perceived Value), a, b, c_1_, c_2_ are the parameters to be estimated, and $\mu_{1}$,$\mu_{2}$, denote the error terms.

(2) Chain mediation effect model

$$L_{i}=a_{1}\cdot Guide_{i}+a_{4}\cdot Controls_{i}+\mu_{4}$$ (6)

$$V_{i}=a_{2}\cdot Guide_{i}+d\cdot L_{i}+\alpha_{5}\cdot Controls_{i}+\mu_{5}$$ (7)

$$Behavior_{i}=c^{\prime }\cdot Guide_{i}+b_{1}\cdot L_{i}+b_{2}\cdot V_{i}+\alpha_{6}\cdot Controls_{i}+\mu_{6}$$ (8)

where *L*_*i*_is mediator 1 (Environmental literacy) and *V*_*2*_is mediator 2 (Perceived Value); *a*_*1*_, *d*, and *b*_*2*_denote the total indirect effect of chained mediated paths; and the rest of the notation is consistent with the above.

## 4 Findings

### 4.1 Direct effects test

Table 4 presents the estimated coefficients and significance levels for the direct effects of Government publicity and guidance, Perceived Value, and Environmental literacy on farmers’ ecological environment governance participation behavior.

**Table 4:** Estimated Direct Effects of Government Publicity and Guidance, Perceived Value, and Environmental Literacy on Farmers’ Ecological Environment Governance Participation Behavior.

Independent Variable	Farmers’ Ecological environment governance participation behavior			Farmers’ Ecological environment governance participation behavior			Farmers’ Ecological environment governance participation behavior		
	Decision-making behavior	Protective behavior	Supervisory behavior	Decision-making behavior	Protective behavior	Supervisory behavior	Decision-making behavior	Protective behavior	Supervisory behavior
Government publicity and guidance	1.552 ^***^	2.301^***^	0.589 ^***^	–	–	–	–	–	–
Environmental Literacy	–	–	–	1.533 ^***^	2.189 ^***^	2.649 ^***^	–	–	–
Perceived Value	–	–	–	–	–	–	2.654 ^***^	2.013 ^***^	2.112 ^***^
Gender	0.19	0.441^*^	−0.042	0.051	−0.171	−0.283^*^	−0.254^*^	−0.183	−0.249^*^
Age	−0.104	0.060	−0.107	0.003	−0.094	−0.141	−0.119	−0.073	−0.118
Health status	0.062	0.222	0.012	0.135^*^	0.229 ^**^	0.091	0.037	0.221^**^	0.106
Education level	0.048	−0.104	0.101	0.219^**^	0.076	0.150	0.338 ^***^	0.151	0.207^*^
Party member	0.456^**^	0.281	0.918 ^***^	0.180	0.021	1.026 ^***^	0.599 ^***^	0.118	1.071 ^***^
Village cadre	1.729 ^***^	−0.001	0395	0.248	1.171 ^**^	0.622	1.940 ^***^	0.767	0.394
Cooperative membership	0.125	−0.324	−0.002	0.020	−0.057	0.061	0.294	0.006	0.161
Cropland quality	0.222^**^	−0.208	0.146^*^	−0.002	−0.112	0.134^*^	−0.009	−0.186^**^	0.034
Agricultural labor force	0.352^**^	0.207	−0.004	−0.104	0.068	−0.038	0.132	0.038	−0.017
Pseudo R^2^	0.443	0.593	0.192	0.038	0.159	0.200	0.216	0.145	0.179
Log likelihood	−217.361	−91.450	−327.691	−808.22	−188.795	−324.502	−305.719	−191.765	−332.917
LR chi2	345.04	265.88	156.11	63.11	71.19	162.49	168.33	65.26	145.66

Notes: *** p < 0.01; ** p < 0.05; * p < 0.10. All models control for demographic and household-level covariates.

(1) The coefficients for the effects of government publicity and guidance on decision-making behavior, protective behavior, and supervisory behavior are 1.552, 2.301, and 0.589, respectively—all of which are statistically significant at the 1% level, thereby supporting Hypothesis H1. These results indicate that government publicity and guidance, as a non-coercive external stimulus, serves as a major driver of farmers’ multidimensional ecological governance behavior. Notably, government publicity and guidance exert the strongest influence on protective behavior, suggesting that the dissemination of ecological knowledge, promotion of green agricultural models, and environmental technology outreach significantly enhances farmers’ willingness and capacity to adopt ecological protection measures in production practices. In terms of decision-making behavior, such publicity efforts help improve farmers’ understanding and recognition of ecological policy values, thereby increasing their initiative in participating in village-level institutional design and governance consultations. By contrast, its effect on Supervisory behavior appears relatively weaker, likely due to insufficient awareness and limited institutional channels for participation, resulting in lower levels of engagement in Supervisory behavior compared to other behavioral dimensions. Overall, government publicity and guidance plays a vital role in fostering farmers’ comprehensive participation in ecological governance, particularly by strengthening daily conservation practices. Meanwhile, the control variables exhibit limited statistical significance, further highlighting the crucial influence of government publicity and guidance. From a political economy perspective, government publicity and guidance effectively transforms national governance intentions into farmers’ cognitive consensus and behavioral self-awareness, thereby promoting farmers’ proactive engagement in decision-making behavior, protective behavior, and supervisory behavior. These findings suggest that government publicity and guidance not only serves as a conduit for policy communication but also plays an essential role in institutional embedding and behavioral activation.
(2) The estimated coefficients for the effects of environmental literacy on farmers’ decision-making behavior, protective behavior, and supervisory behavior were 1.533, 2.189, and 2.649, respectively, all statistically significant at the 1% level, thus providing strong support for Hypothesis H2. The results suggest that environmental literacy, as a critical intra-individual cognitive factor, exerts a significant and positive influence on farmers’ multidimensional ecological and environmental governance behaviors. The strongest effect of environmental literacy was observed for supervisory behavior, indicating that farmers with higher environmental literacy are more capable and willing to engage in governance monitoring by identifying problems and actively providing feedback during the implementation of ecological governance initiatives. With respect to protective behavior, environmental literacy enhances farmers’ understanding of sustainable agricultural technologies and environmental conservation practices, increasing their willingness to incorporate environmental protection concepts into daily agricultural practices and strengthening their commitment to ecological protection. In the context of decision-making behavior, environmental literacy improves farmers’ awareness and recognition of ecological policy objectives, thereby encouraging more active participation in the design of village-level governance systems and the negotiation of ecological rules. Overall, environmental literacy significantly promotes farmers’ engagement in ecological governance and plays a particularly prominent role in strengthening supervisory behavior, underscoring its central function in enhancing farmers’ governance capacity and environmental responsibility.
(3) The estimated coefficients for the effects of Perceived Value on farmers’ Decision-making behavior, Protective behavior, and Supervisory behavior were 2.654, 2.013, and 2.112, respectively, all statistically significant at the 1% level, thus providing robust support for Hypothesis H3. These results indicate that Perceived Value, as a key psychological and cognitive factor influencing farmers’ decision-making behavior, exerts a significant and positive influence on promoting their multidimensional ecological environment governance participation behavior. Among the three behavioral dimensions, Perceived Value had the strongest effect on Decision-making behavior, suggesting that when farmers perceive ecological governance as a source of multiple benefits—including economic returns, social recognition, and environmental enhancement—they are more likely to actively engage in public decision-making activities such as policy endorsement and village governance rule-making. In the case of Supervisory behavior, a higher level of Perceived Value encourages farmers to place greater emphasis on governance effectiveness and motivates them to engage more actively in project supervision and feedback processes to ensure both policy performance and protection of their own interests. The relatively lower effect of Perceived Value on Protective behavior may be attributed to the fact that protective behaviors often involve greater resource demands and physical effort, which may constrain the extent to which perceived value is translated into concrete actions. Overall, Perceived Value exerts a comprehensive and powerful influence on farmers’ ecological governance behavior, particularly by enhancing their policy recognition and behavioral motivation, highlighting the intrinsic motivational function of Perceived Value in shaping ecological engagement among farmers.

### 4.2 Robustness type test

In order to verify the robustness of the regression results, two robustness checks were conducted by replacing the dependent variables and estimation models, respectively (see Table 5). First, the original dependent variables—namely, the number of farmers’ Decision-making behavior, Protective behavior, and Supervisory behavior—were converted into binary variables indicating whether the farmer had participated in each respective activity during the survey period. That is, a value of 1 was assigned if the farmer engaged in at least one relevant activity, and 0 otherwise. The regression results show that government publicity and guidance, environmental literacy, and perceived value still exert significant and positive influences on farmers’ ecological environment governance participation behavior, with both the direction and significance levels remaining consistent with the baseline findings. Second, the core relationships were re-estimated using an OLS linear regression model, and the results remained robust. Overall, the empirical results remained qualitatively unchanged under both model and variable substitutions, thereby confirming the robustness and reliability of the study’s conclusions.

**Table 5:** Robustness Tests.

Type of Test	Independent Variable	Ecological Environment Governance Participation Behavior			Ecological Environment Governance Participation Behavior			Ecological Environment Governance Participation Behavior		
		Decision-Making Behavior	Protective Behavior	Supervisory Behavior	Decision-Making Behavior	Protective Behavior	Supervisory Behavior	Decision-Making Behavior	Protective Behavior	Supervisory Behavior
Substitution of Variables	Government publicity and guidance	2.533 ^***^	2.283 ^***^	0.395 ^***^	–	–	–	–	–	–
	Environmental Literacy	–	–	–	2.115 ^***^	2.184 ^***^	2.495 ^***^	–	–	–
	Perceived Value	–	–	–	–	–	–	1.965 ^***^	2.085 ^***^	1.926 ^***^
	Control Variable	Controlled	Controlled	Controlled	Controlled	Controlled	Controlled	Controlled	Controlled	Controlled
Replacement of Model	Government publicity and guidance	0.882 ^***^	1.638 ^***^	0.583 ^***^	–	–	–	–	–	–
	Environmental Literacy	–	–	–	1.875 ^***^	2.975 ^***^	1.959 ^***^	–	–	–
	Perceived Value	–	–	–	–	–	–	2.272 ^***^	2.859 ^***^	1.961 ^***^
	Control Variable	Controlled	Controlled	Controlled	Controlled	Controlled	Controlled	Controlled	Controlled	Controlled

Notes: *** p < 0.01; ** p < 0.05; * p < 0.10. All models control for demographic and household-level covariates.

### 4.3 Heterogeneity test

The income level of farming households directly influences their access to resources, capacity for risk tolerance, and responsiveness to policy initiatives. Consequently, their decision-making behavior may exhibit notable heterogeneity. To examine whether the core findings of this study hold across different household income groups, the sample was stratified into high-income and low-income subgroups based on per capita annual household income. A heterogeneity analysis was then conducted, with the results presented in Table 6. The findings reveal that government publicity and guidance, environmental literacy, and perceived value all exert significant positive effects on farmers’ ecological environment governance participation behavior in both income groups. This suggests that the study’s conclusions possess broad applicability and explanatory robustness across heterogeneous populations, thereby validating the generalizability and consistency of the empirical results.

**Table 6:** Heterogeneity test.

independent variable	Decision-Making Behavior		Protective Behavior		Supervisory Behavior	
	Low-income farmers	High-income farmers	Low-income farmers	High-income farmers	Low-income farmers	High-income farmers
Government publicity and guidance	0.183 ^***^	0.157 ^***^	0.348 ^***^	0.372 ^***^	0.131 ^***^	0.111 ^***^
environmental literacy	0.525 ^***^	0.488 ^***^	0.548 ^***^	0.535 ^***^	0.594 ^***^	0.475 ^***^
Perceived Value	0.514 ^***^	0.492 ^***^	0.537 ^***^	0.533 ^***^	0.554 ^***^	0.437 ^***^
Log likelihool	−98.714	−116.196	−37.909	−49.897	−139.27	−183.001
control variable	Controlled	Controlled	Controlled	Controlled	Controlled	Controlled
sample size	157	158	157	158	157	158

Notes: *** p < 0.01; ** p < 0.05; * p < 0.10. All models control for demographic and household-level covariates.

Further comparison of the grouped regression coefficients reveals that the three core variables—Government publicity and guidance, Environmental literacy, and Perceived Value—each correspond to farmers’ Decision-making behavior, Protective behavior, and Supervisory behavior, forming a total of nine behavioral influence pathways. Among these nine paths, only the effect of government publicity and guidance on protective behavior is significantly stronger among high-income farmers than low-income farmers. In the remaining eight paths, the coefficients are consistently higher in the low-income group, suggesting that low-income farmers exhibit greater behavioral sensitivity to government publicity and guidance, environmental literacy, and perceived value, thereby highlighting distinct heterogeneity in behavioral responses. Several potential explanations account for these findings. First, regarding government publicity and guidance, low-income farmers tend to have limited access to information, technology, and resources, making them more reliant on the financial subsidies, project assistance, and technical services emphasized in government communications. Consequently, policy messages are transmitted more directly and elicit stronger behavioral responses. In contrast, high-income farmers often possess diversified access to external resources and information, diminishing the marginal incentive effect of government publicity and guidance. Second, with respect to environmental literacy, low-income groups typically start from a lower baseline of environmental knowledge and skills. As a result, educational interventions and policy training produce more substantial cognitive gains, thereby significantly enhancing their willingness and capacity to engage in ecological governance. Conversely, high-income farmers, having already accumulated a moderate level of environmental awareness, experience diminishing returns from additional literacy improvements. Third, in terms of Perceived Value, Low-income farmers are more responsive to the tangible economic, social, and environmental benefits of ecological governance. Their participation is more strongly motivated by practical considerations, such as improvements in land quality, access to subsidies, or enhancements in the local living environment. In contrast, high-income farmers are less driven by immediate material gains and more by long-term environmental awareness and a heightened sense of social responsibility.

### 4.4 Indirect effects test

According to the previous theoretical analysis, in addition to the direct effect of government publicity and guidance on farmers’ ecological environment governance participation behavior, it may also have an indirect effect through the two internal variables of environmental literacy and perceived value. Based on this, this study utilizes the mediation effect model and introduces the bootstrap test to test the above mediation effects, and the results are shown in Table 7. The results show that: (1) government publicity and guidance significantly affects farmers’ ecological environmental governance behavior through the mediation path of environmental literacy, and hypothesis H4 is valid. This indicates that the quantity and quality of environmental protection information provided to farmers by the Government publicity and guidance through ecological governance can directly affect the depth and breadth of farmers’ understanding of environmental issues, deepen their understanding of the functions of the ecosystem, the need for ecological balance and the urgency of sustainable development, and enhance their environmental literacy, thereby strengthening their motivation to participate in decision-making, implementation of protection, and supervision of governance behavior. The government publicizes and guides farmers through the perception of ecosystem function and the need for ecological balance and the urgency of sustainable development. (2) The mediation path of government publicity and guidance influencing farmers’ ecological environment governance participation behavior through perceived value is also significant, and hypothesis H5 is valid. The hypothesis H5 is also significant, indicating that farmers can form a positive perception of the economic return, social recognition and environmental improvement of ecological governance under the influence of government publicity, which in turn enhances their motivation and behavioral response to participate in ecological governance. (3) Environmental literacy and guidance and Perceived Value play a chain mediating path between government publicity and guidance and farmers’ ecological environment governance participation behavior. That is, government publicity and guidance first enhance environmental literacy, then enhance farmers’ perceived value of ecological governance, and ultimately indirectly promote their participation in ecological governance behavior, reflecting the role of environmental literacy as a bridge between information absorption and value construction.

**Table 7:** Indirect effect test of Environmental literacy, Perceived Value in Government publicity and guidance in influencing farmers’ Ecological environment governance participation behavior.

independent variable	Environmental literacy intermediary test				Perceived Value Mediation Tests				Environmental literacy, Perceived Value Chained Mediation Tests			
	Environmental literacy	Ecological environment governance participation behavior			Perceived Value	Ecological environment governance participation behavior			Perceived Value	Ecological environment governance participation behavior		
		Decision-making behavior	Protective behavior	Supervisory behavior		Decision-making behavior	Protective behavior	Supervisory behavior		Decision-making behavior	Protective behavior	Supervisory behavior
Government publicity and guidance	0.067 ^***^	0.812^***^	1.546 ^***^	0.485 ^***^	0.073 ^***^	0.781 ^***^	1.051 ^***^	0.479 ^***^	0.062 ^***^	0.737 ^***^	1.495 ^***^	0.413 ^***^
environmental literacy	–	1.031 ^***^	1.370 ^***^	1.455 ^***^	–	–	–	–	0.180 ^***^	0.812^***^	1.222 ^***^	1.244 ^***^
Perceived Value	–	–	–	–	–	1.367 ^***^	1.561 ^***^	1.405 ^***^	–	1.216 ^***^	0.823 ^***^	1.173 ^***^
control variable	Controlled	Controlled	Controlled	Controlled	Controlled	Controlled	Controlled	Controlled	Controlled	Controlled	Controlled	Controlled

Notes: *** p < 0.01; ** p < 0.05; * p < 0.10. All models control for demographic and household-level covariates.

## 5 Discussion

Farmers’ ecological environment governance participation behavior serves as a pivotal element in achieving agricultural green transformation and rural ecological revitalization. It underpins the effectiveness of grassroots ecological policies and the implementation of sustainable development strategies. Unlike existing studies that predominantly focus on a single behavioral dimension—such as policy adoption or decision-making behavior—this study proposes a more integrative framework encompassing decision-making behavior, protective behavior, and supervisory behavior, and systematically delineates the formation pathways of farmers’ multi-dimensional behavioral responses. From a theoretical perspective, this study draws upon SOR (Stimulus-Organism-Response) theory to integrate external policy stimuli and internal cognitive mechanisms. By incorporating environmental literacy and perceived value as mediating variables, it constructs a chain mediation model of “government publicity and guidance → cognitive structure → behavioral response.” This model enriches the theoretical understanding of the behavioral mechanisms underlying agro-ecological governance and provides a novel analytical approach to evaluating the behavioral effects of non-coercive policy instruments.

This study reaffirms prior findings that government publicity and guidance significantly promotes farmers’ protective behavior [37]. Moreover, the results extend prior findings by demonstrating that government publicity and guidance also exerts a significant influence on farmers’ decision-making behavior and supervisory behavior, thereby broadening the understanding of its impact beyond single-dimensional ecological responses. In parallel, both Environmental literacy and Perceived Value were found to significantly influence all three behavioral dimensions, functioning as key internal mechanisms through which external policy stimuli are transformed into concrete ecological governance actions by farmers [26,27]. The heterogeneity analysis further revealed that these three core factors had more pronounced effects on the behavioral responses of low-income farmers, indicating that policy publicity and perceived incentives produce higher marginal impacts among resource-constrained groups. Mediation analysis confirmed that both Environmental literacy and Perceived Value serve as significant mediators between government publicity and guidance and farmers’ behavioral responses, and jointly constitute a chain mediation effect that highlights the pivotal role of farmers’ cognitive structures in translating policy signals into ecological governance outcomes [21].

These findings contribute to elucidating the psychological logic and behavioral response mechanisms underlying farmers’ ecological environment governance participation behavior. Nonetheless, this study has certain limitations that warrant consideration. First, the data were primarily collected from Changji City, Xinjiang, which—despite its representative ecological governance challenges and household structure—may limit the external generalizability of the findings to other agricultural contexts. Second, the measurement of farmers’ ecological behaviors and cognitive variables primarily relied on self-reported questionnaires. Although model robustness tests were conducted to improve the reliability of the results, it remains difficult to fully eliminate potential social desirability bias. Future research could incorporate field interviews, behavioral tracking, and policy intervention experiments to enhance data objectivity and deepen explanatory power.

## 6 Conclusions and Implications

This study empirically examines the effects of government publicity and guidance on farmers’ multi-dimensional ecological environment governance participation behavior and its internal mechanism of action based on rural micro-research data in Changji Prefecture, Xinjiang, using an ordered probit model, with the following main findings: (1) Government publicity and guidance, environmental literacy and perceived value have a significant positive effect on farmers’ decision-making behavior, protective behavior and supervisory behavior. This conclusion is still firmly established after the robustness test using alternative variables and changing model settings. (2) Sub-group heterogeneity analysis shows that government publicity and guidance, environmental literacy and perceived value have stronger promotional effects on the ecological environment governance participation behavior of low-income farmers, indicating that compared with the high-income group, low-income farmers have stronger marginal effects on the behavioral response to policy guidance and cognitive incentives, and that the effect of external intervention is more significant. (3) Mechanism analysis reveals that Environmental literacy and Perceived Value both play a significant mediating role in the process of government publicity and guidance influencing farmers’ ecological environment governance participation behavior, and two together construct a chain mediation path of “government publicity and guidance-environmental literacy-perceived value-ecological and environmental governance behavior”.

Based on the above findings, this study provides the following policy recommendations:(1) Refine government publicity and guidance strategies to enhance targeting. Publicity efforts should be designed with clear objectives and tailored content, emphasizing the promotion of farmers’ awareness of ecological governance measures. Through professional training, on-site demonstrations, and practical case studies, governments should improve the reach and precision of publicity efforts. This requires a shift from traditional “policy indoctrination” toward “cognitive stimulation” to increase farmers’ policy recognition and willingness to participate.(2) Systematically enhance farmers’ environmental literacy. Environmental education should be considered foundational to ecological governance. Governments can organize rural environmental protection workshops, eco-agricultural technical training sessions, and green production demonstration activities to comprehensively improve farmers’ ecological knowledge, environmental awareness, and capacity for sustainable practices, thereby strengthening their intrinsic motivation to participate actively in governance.(3) Facilitate the internalization of farmers’ Perceived Value. Efforts should be made to deepen farmers’ internal understanding of the significance of ecological governance. This can be achieved by using positive publicity and educational initiatives—such as presenting successful cases and model projects—to demonstrate the long-term benefits of ecological governance for both community welfare and individual well-being.(4) Implement differentiated policy support mechanisms. Given that low-income farmers are more responsive to policy publicity and perception-driven incentives, resources and support should be strategically allocated to this group. Targeted subsidies, customized services, and inclusive participation platforms should be established to encourage their deeper engagement in ecological governance and to ensure the differentiated implementation of policy impacts across income groups.

## Supporting information

S1 File Minimal data set. (XLSX)

S2 File Supplementary form. (DOCX)
