# Supplementary material for: The effect of government publicity and guidance on farmers’ ecological environment governance participation behavior—The mediating effect of environmental literacy and perceived value
Source: PLoS One. 2025 Jul 23;20(7):e0328274. doi: 10.1371/journal.pone.0328274 (PMC12286404; doi:10.1371/journal.pone.0328274)
Supplement: S2 File — (DOCX) [file pone.0328274.s002.docx]

**Robustness type test**

**Robustness Tests**（Substitution of Variables）

| Independent Variable | Decision-Making Behavior | Protective Behavior | Supervisory Behavior | Decision-Making Behavior | Protective Behavior | Supervisory Behavior | Decision-Making Behavior | Protective Behavior | Supervisory Behavior |
| --- | --- | --- | --- | --- | --- | --- | --- | --- | --- |
| Government publicity and guidance | 2.533^***^ | 2.283^***^ | 0.395^***^ | - | - | - | - | - | - |
| Environmental Literacy | - | - | - | 2.115^***^ | 2.184^***^ | 2.495^***^ | - | - | - |
| Perceived Value | - | - | - | - | - | - | 1.965^***^ | 2.085^***^ | 1.926^***^ |
| Control Variable | Controlled | Controlled | Controlled | Controlled | Controlled | Controlled | Controlled | Controlled | Controlled |
| Pseudo R^2^ | 0.663 | 0.601 | 0.196 | 0.186 | 0.163 | 0.250 | 0.178 | 0.154 | 0.215 |
| Log likelihool | -73.606 | -87.105 | -175.045 | -177.638 | -182.764 | -163.202 | -179.499 | -184.792 | -170.836 |
| LR chi2 | 289.47 | 262.47 | 85.19 | 81.40 | 71.15 | 108.88 | 77.68 | 67.10 | 93.61 |

**Robustness Tests**（Replacement of Model）

| Independent Variable | Decision-Making Behavior | Protective Behavior | Supervisory Behavior | Decision-Making Behavior | Protective Behavior | Supervisory Behavior | Decision-Making Behavior | Protective Behavior | Supervisory Behavior |
| --- | --- | --- | --- | --- | --- | --- | --- | --- | --- |
| Government publicity and guidance | 0.882^***^ | 1.638^***^ | 0.583^***^ | - | - | - | - | - | - |
| Environmental Literacy | - | - | - | 1.875^***^ | 2.975^***^ | 1.959^***^ | - | - | - |
| Perceived Value | - | - | - | - | - | - | 2.272^***^ | 2.859^***^ | 1.961^***^ |
| Control Variable | Controlled | Controlled | Controlled | Controlled | Controlled | Controlled | Controlled | Controlled | Controlled |
| Constant | -2.010*** | -2.810*** | -0.998* | -0.906* | -0.532 | -0.589 | -0.706 | -0.005 | -0.263 |
| R-squared | 0.693 | 0.577 | 0.459 | 0.463 | 0.198 | 0.423 | 0.506 | 0.189 | 0.422 |

**Heterogeneity test**

| independent variable | Decision-Making Behavior | | Protective Behavior | | Supervisory Behavior | |
| --- | --- | --- | --- | --- | --- | --- |
|  | Low-income farmers | High-income farmers | Low-income farmers | High-income farmers | Low-income farmers | High-income farmers |
| Government publicity and guidance | 0.183 ^***^ | 0.157 ^***^ | 0.348 ^***^ | 0.372 ^***^ | 0.131 ^***^ | 0.111 ^***^ |
| control variable | Controlled | Controlled | Controlled | Controlled | Controlled | Controlled |
| Pseudo R^2^ | 0.458 | 0.4335 | 0.650 | 0.565 | 0.222 | 0.175 |
| Log likelihool | -98.714 | -116.196 | -37.909 | -49.897 | -139.27 | -183.001 |
| LR chi2 | 167.06 | 177.86 | 141.06 | 129.46 | 79.61 | 77.65 |
| sample size | 157 | 158 | 157 | 158 | 157 | 158 |

| independent variable | Decision-Making Behavior | | Protective Behavior | | Supervisory Behavior | |
| --- | --- | --- | --- | --- | --- | --- |
|  | Low-income farmers | High-income farmers | Low-income farmers | High-income farmers | Low-income farmers | High-income farmers |
| Perceived Value | 0.514 ^***^ | 0.492 ^***^ | 0.537 ^***^ | 0.533 ^***^ | 0.554 ^***^ | 0.437 ^***^ |
| control variable | Controlled | Controlled | Controlled | Controlled | Controlled | Controlled |
| Pseudo R^2^ | 0.225 | 0.228 | 0.199 | 0.147 | 0.255 | 0.144 |
| Log likelihool | -141.319 | -158.322 | -86.905 | -97.779 | -133.38 | -189.98 |
| LR chi2 | 81.85 | 93.61 | 43.07 | 33.69 | 91.38 | 63.69 |
| sample size | 157 | 158 | 157 | 158 | 157 | 158 |

| independent variable | Decision-Making Behavior | | Protective Behavior | | Supervisory Behavior | |
| --- | --- | --- | --- | --- | --- | --- |
|  | Low-income farmers | High-income farmers | Low-income farmers | High-income farmers | Low-income farmers | High-income farmers |
| environmental literacy | 0.525 ^***^ | 0.488 ^***^ | 0.548 ^***^ | 0.535 ^***^ | 0.594 ^***^ | 0.475 ^***^ |
| control variable | Controlled | Controlled | Controlled | Controlled | Controlled | Controlled |
| Pseudo R^2^ | 0.239 | 0.198 | 0.217 | 0.149 | 0.267 | 0.163 |
| Log likelihool | -138.67 | -164.480 | -84.908 | -97.599 | -131.290 | -185.745 |
| LR chi2 | 87.15 | 81.29 | 47.06 | 34.05 | 95.57 | 72.16 |
| sample size | 157 | 158 | 157 | 158 | 157 | 158 |
